# A Rapid Detection of *Haemophilus influenzae* Using Multiple Cross Displacement Amplification Linked With Nanoparticle-Based Lateral Flow Biosensor

**DOI:** 10.3389/fcimb.2021.721547

**Published:** 2021-09-22

**Authors:** Qilong Cao, Shaoshuai Liang, Lin Wang, Jun Cao, Mengyang Liu, Shengpeng Li, Xiaolong Cao, Yan Guo

**Affiliations:** ^1^ Biomedical Informatics & Genomics Center, Key Laboratory of Biomedical Information Engineering of Ministry of Education, School of Life Science and Technology, Xi'an Jiaotong University, Xi’an, China; ^2^ Research and Development Department, Qingdao Haier Biotech Co. Ltd, Qingdao, China; ^3^ Hefei National Laboratory for Physical Sciences at the Microscale, School of Life Sciences, Division of Life Sciences and Medicine, University of Science and Technology of China, Hefei, China; ^4^ Department of Clinical Laboratory, The Affiliated Hospital of Qingdao University, Qingdao, China; ^5^ Department of Clinical Laboratory, Qingdao Youfu Hospital, Qingdao, China; ^6^ Outpatient Department, Beijing Changping Institute for Tuberculosis Prevention and Treatment, Beijing, China

**Keywords:** *Haemophilus influenzae*, MCDA-LFB, nanoparticle-based biosensor, rapid detection, potential diagnostic tool

## Abstract

*Haemophilus influenzae* is a major human pathogenic bacterium, resulting in a series of diseases, such as pneumonia, bacteremia, meningitis. However, it is hard to diagnose *H*. *influenzae* quickly. In this study, the multiple cross displacement amplification (MCDA) and nanoparticle-based lateral flow biosensor (LFB) (MCDA-LFB) were combined to detect *H*. *influenzae*, which has been proven to be reliable, rapid, and not complicated. On the basis of *H*. *influenzae* outer membrane protein *P6* gene, 10 specific primers were designed. The best MCDA condition was 61°C for 1 h. The sensitivity of *H*. *influenzae*-MCD-LFB assay showed, in the pure cultures, the minimum concentration of genomic DNA templates was 100 fg. The specificity of *H*. *influenzae*-MCD-LFB assay showed only *H*. *influenzae* templates were detected, and no cross-reactivity was found in non-*H*. *influenzae* isolates and other *Haemophilus* species. In 56 sputum samples, with MCDA-LFB method and PCR detection, 21 samples were positive, which was in consistent with the traditional culture method. The accuracy of diagnosis of MCDA-LFB, in comparison with the traditional culture method and PCR detection, can reach 100%, indicating that the MCDA-LFB assay gains an advantage over the cultured-based method for target pathogen detection. In conclusion, the MCDA-LFB assay is suitable for the sensitive, rapid, and specific detection of *H*. *influenzae*, which might be used as a potential diagnostic tool for *H*. *influenzae* in basic and clinical laboratories.

## Introduction

*Haemophilus influenzae* (*H. influenzae*), a kind of Gram-negative bacterium, is an important human pathogen (Deich et al., 1988). Non-typical *H. influenzae* has been proven to be involved in numerous diseases, such as pneumonia, bacteremia, acute febrile tracheobronchitis in adults (Murphy et al., 1985; Deich et al., 1988), and resulted in 20 to 40% of otitis media in children (Howie et al., 1970; Deich et al., 1988). *H. influenzae* type b (Hib) can lead to pneumonia, epiglottitis, and meningitis predominantly in infants and young children (Peltola, 2000; Kim et al., 2011). In many countries, the introduction of Hib conjugate vaccines into routine immunization has significantly decreased the morbidity of disease associated with Hib, which confirmed the effectiveness of the traditional antimicrobial therapy (Barbour, 1996; Kim et al., 2011). Therefore, the researches in isolation of antimicrobial-resistant *H. influenzae* strains have increased rapidly (Hasegawa et al., 2004; Torigoe et al., 2007). Currently, because of the difficulty of distinguishing *H. influenzae* from closely related *Haemophilus* spp., for example, *Haemophilus parainfluenzae* (*H. parainfluenzae*), the process of isolating and identifying *H. influenzae* strains are time-consuming and complicated (Torigoe et al., 2007). Moreover, the classical techniques are also time-consuming and complicated, for instance, colonial morphology, growth-based assays, and serological determination (Torigoe et al., 2007). Nevertheless, the traditional techniques were still used in a large number of diagnostic laboratories. In order to realize early diagnosis and effective antibiotic therapy, the sensitive and specific methods are essential to be established (Brown and Lerner, 1998; Torigoe et al., 2007). 

Based on the PCR assays, with the primers specific for rRNA-encoding genes, the *H. influenzae* detection has achieved a measure of success (Hendolin et al., 1997; Lu et al., 2000). However, due to the about 95% homology of rRNA sequences between *H. influenzae* and *H. parainfluenzae*, the rRNA-encoding genes are not suitable for the accurate identification of *H. influenzae*. It has been reported that the capsulation-associated protein Bex A gene and the outer membrane protein (OMP) P6 gene could be specific diagnostic targets (van Ketel et al., 1990). Whereas the primers based on Bex A protein gene could merely react with capsulated strains of *H. influenzae*, but did not react with non-typical *H. influenzae* strains (Torigoe et al., 2007). OMP P6 is highly conservative among a majority of strains of *H. influenzae* (Murphy et al., 1986; Nelson et al., 1991), which might be a potential vaccine component used to prevent the infection by *H. influenzae* (Karalus and Murphy, 1999). The specific OMP *P6* gene of *H. influenzae* is well characterized (Nelson et al., 1988), and some studies have been made to detected *H. influenzae* with this gene (van Ketel et al., 1990; Hotomi et al., 1993; Ueyama et al., 1995). Besides, for the diagnosis of Hib in the laboratory, the PCR used to detect *bexA* (*bexA* PCR) (Corless et al., 2001) and a nested PCR used to detect Hib (Hib PCR) (Falla et al., 1994) have been an important milestone. It is unfortunate that the PCR-based methods are expensive and complex for resource-limited laboratory settings.

It has been reported that multiple cross displacement amplification (MCDA, Chinese IP Office Patent Application CN20150280765.X) could be applied to detect specific nucleic-acid sequences, as a replacement for PCR-based method (Wang et al., 2015). Ten primers designed for binding to the 10 regions of target sequences are required in the MCDA assay to achieve the specific amplification in a temperature range from 60 to 67°C. The advantages of the MCDA technique seemed to be rapidity, sensitivity, and specificity. Particularly, the nanoparticle-based lateral flow biosensor (LFB) has been used to present the results of MCDA (MCDA-LFB assay), which only takes about 2 min and is easy to use (Wang et al., 2016; Wang et al., 2018). In addition, the coronavirus disease 19 (COVID-19) also has been diagnosed with a reverse transcription multiple cross displacement amplification (RT-MCDA) linked with nanoparticles-based biosensor (BS) assay (RT-MCDA-BS), which greatly improved the convenience (Li et al., 2020).

In this study, we firstly report an MCDA coupled with LFB (MCDA-LFB) method for rapid, simple, and reliable detection of *H. influenzae* based on target sequence and validate its potential clinical application with clinical samples.

## Materials and Methods

### Reagents and Instruments

FastPure® Blood/Cell/Tissue/Bacteria DNA Isolation Mini Kit was purchased from Vazyme Biotech Co., Ltd (Nanjing, China). Isothermal amplification kit, Visual Detection Reagent (VDR), and Nanoparticle-based lateral flow biosensor were purchased from BeiJing-HaiTaiZhengYuan Technology Co., Ltd (Beijing, China). The specific primers used in this study were synthesized by TSINGKE Biological Technology Co., Ltd (Beijing, China). LA-320C Real-time Turbidimeter was purchased from Eiken Chemical Co., Ltd (Tokyo, Japan).

### Bacterial Strains and Genomic Template Preparation

In total, 28 clinical isolated strains, involving 7 *H. influenzae* strains, 17 non-*H. influenzae* strains, and other *Haemophilus* species, such as 2 *Haemophilus parainfluenzae* strains, 1 *Haemophilus haemolyticus* strain, and 1 *Haemophilus parahaemolyticus* strain, were used in this study (**Table 1**). The genomic DNA templates were extracted with FastPure® Blood/Cell/Tissue/Bacteria DNA Isolation Mini Kit (Nanjing, China) according to the manufacturer’s instructions. The templates were quantified with ultraviolet spectrophotometer (NanoDrop One, Thermo, USA) at A260/280 and stored under −20°C before use. An isolate of *H. influenzae* designed as reference strain was applied in the optimization and sensitivity analysis with pure culture.

**Table 1:** Bacterial strains used in this study.

Bacteria species	Isolates (source)	No. of strains
*Haemophilus influenzae*	Isolates	7
Enteroadhesive *E. coli*	Isolates	1
Enterohemorrhagic *E. coli*	Isolates	1
Enteropathogenic *E. coli*	Isolates	1
Enteroinvasive *E. coli*	Isolates	1
Enterotoxigenic *E. coli*	Isolates	1
*Streptococcus suis*	Isolates	1
*Staphylococcus epidermidis*	Isolates	1
*Staphylococcus haemolyticus*	Isolates	1
*Acinetobacter baumannii*	Isolates	1
*Listeria monocytogenes*	Isolates	2
*Pseudomonas aeruginosa*	Isolates	2
*Enterococcus faecalis*	Isolates	1
*Staphylococcus aureus*	Isolates	1
*Klebsiella pneumoniae*	Isolates	2
*Haemophilus parainfluenzae*	Isolates	2
*Haemophilus haemolyticus*	Isolates	1
*Haemophilus parahaemolyticus*	Isolates	1

### Primer Design 

**Figure 1:**
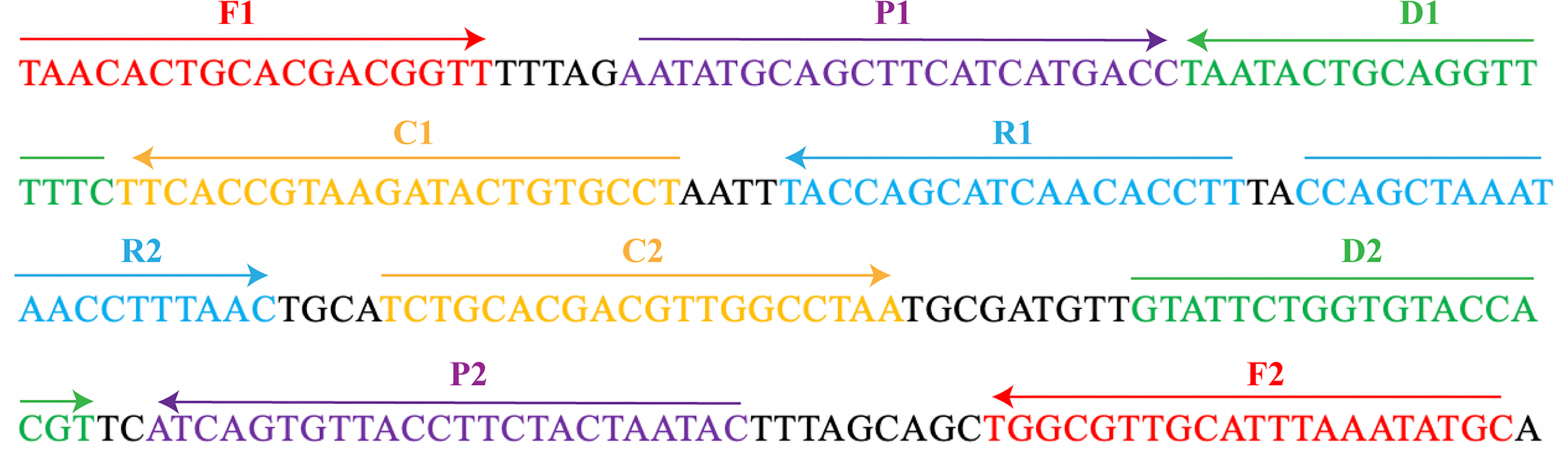
The MCDA primers designed based on the OMP *P6* gene. The location of the 10 primers were marked with different colors. Right arrows and left arrows indicated sense and complementary sequences.

The MCDA primers were designed targeted to OMP *P6* gene (Genbank accession no. L42023) of the *H. influenzae* with PRIMER 5.0 software and PrimerExplorer V4 (http://primerexplorer.jp/elamp4.0.0/index.html). The hairpin structures and hybrids of the 10 primers, involving F1 and F2 two displacement primers, CP1 and CP2 two cross primers, and C1, C2, D1, D2, R1, and R2 six amplification primers, were analyzed with the Integrated DNA Technologies design tool (http://www.idtdna.com/site). The specificity of MCDA primers for *H. influenzae* was verified by blast analysis. To detect MCDA products with LFB, the 5’- of C1 and 5’- of D1 primers were respectively labeled with FAM and biotin. All primers synthesized by TSINGKE Biological Technology Co., Ltd. (Beijing, China) at HPLC purification grade are listed in **Table 2**. The primer sequences and locations are shown in **Figure 1**.

**Table 2:** The primers used in this study.

Primers	Sequences and modifications (5’-3’)	Length	Gene
F1	TAACACTGCACGACGGTT	18 nt	
F2	GCATATTTAAATGCAACGCCA	21 nt	
CP1	AGGCACAGTATCTTACGGTGAA-		
	AATATGCAGCTTCATCATGACC	45 mer	
CP2	TCTGCACGACGTTGGCCTAA-		
	GTATTAGTAGAAGGTAACACTGAT	45 mer	
C1	AGGCACAGTATCTTACGGTGAA	22 nt	OMP *P6*
C1*	FAM-AGGCACAGTATCTTACGGTGAA	22 nt	
C2	TCTGCACGACGTTGGCCTAA	20 nt	
D1	GAAAAACCTGCAGTATTA	18 nt	
D1*	Biotin-GAAAAACCTGCAGTATTA	18 nt	
D2	GTATTCTGGTGTACCACGT	19 nt	
R1	AAGGTGTTGATGCTGGTA	18 nt	
R2	CCAGCTAAATAACCTTTAAC	20 nt	
PCR F	AACTTTTGGCGGTTACTCTG	20 nt	
PCR R	CTA ACACTGCACGACGGTTT	20 nt	

C1*, 5’-labeled with FAM when used in MCDA-LFB assay; D1*, 5’-labeled with Biotin when used in MCDA-LFB assay.

nt, nucleotide; mer, monomeric.

### The Standard MCDA Assay

The reaction system of MCDA assay was conducted in the mixtures of 25 ul, involving 0.4 μM each of displacement primers F1 and F2, 1.6 μM each of cross primers CP1 and CP2, 0.8 μM each of amplification primers C1*, C2, R1, R2, D1*, and D2, 12.5 μl 2× reaction mix of Isothermal amplification kit (Beijing, China), and 1.25 μl 10 U Bst DNA polymerase, 1 μl DNA template, and 1 μl VDR (Wang et al., 2015). Besides, 1 μl DNA extracted from non-*H. influenzae* was selected as negative control, and 1 μl double-distilled water was as blank control. The amplification reactions were performed at 63°C for 1 h. After that, the reactions were heated at 95°C for 5 min to terminate. Then amplification products were detected by colorimetric indicator and LFB.

### The Optimal Reaction Temperature of MCDA Assay 

*H. influenzae*-MCDA-LFB assay was performed at varying temperatures (58–65°C with 1°C intervals) for 1 h to acquire the optimal reaction temperature, and the amplification processes were monitored by Real-time turbidimeter.

### Specificity and Sensitivity of the *H. influenzae*-MCDA-LFB Assay

To analyze the sensitivity of the MCDA-LFB method, the DNA templates extracted from *H. influenzae* were diluted in a gradient (10 ng, 1 ng, 100 pg, 10 pg, 1 pg, 100 fg, 10 fg, and 1 fg per μl). Then, the 1 μl of each diluted template were used for the MCDA-LFB assay. The limit of detection (LoD) of MCDA-LFB assay was defined by genomic DNA amount of the template. Three replicates of each dilution were examined.

The specificity of *H. influenzae*-MCDA-LFB was confirmed with DNA templates extracted from 7 *H. influenzae* strains, 17 non-*H. influenzae* strains, 2 *H. parainfluenzae* strains, 1 *H. haemolyticus* strain, and 1 *H. parahaemolyticus* strain (**Table 1**). The assays were repeated at least twice.

### *H. influenzae-*MCDA-LFB Assay in Clinical Samples

To evaluate the practicability of *H. influenzae-*MCDA-LFB assay in clinical sample detection, we collected sputum samples of patients who were suspected of infecting with *H. influenzae* in Dingzhou People’s Hospital through three consecutive months. All the sputum samples were detected using traditional culture methods, including Gram stain, colony morphology, and biochemical identification, which were accomplished by Dingzhou People’s Hospital. A total of 21 sputum samples were successfully detected with *H. influenzae* isolates. The 21 *H. influenzae* positive sputum samples and another 35 randomly selected *H. influenzae* negative sputum samples were used to extract genomic DNA using FastPure® Blood/Cell/Tissue/Bacteria DNA Isolation Mini Kit (Nanjing, China) as previously introduced. All the 56 DNA templates were randomly mixed, and *H. influenzae-*MCDA-LFB assay and PCR detection were used to detect *H. influenzae.* The PCR reaction system involving 10 μl 2×T3 super PCR mix of TSINGKE Biological Technology Co., Ltd. (Beijing, China), 7 μl double-distilled water, 1 μl DNA template, and PCR forward (PCR F) and reverse (PCR R) primers (Ueyama et al., 1995) are listed in **Table 2**. The condition of PCR was as follows: 95°C 5 min, followed by 32 cycles of 95°C 30 s, 50°C 30 s, 72°C 30 s. Then the PCR detection and MCDA-LFB detection results were compared with that of traditional culture.

## Results

### Confirmation of *H. influenzae-*MCDA-LFB Products

**Figure 2:**
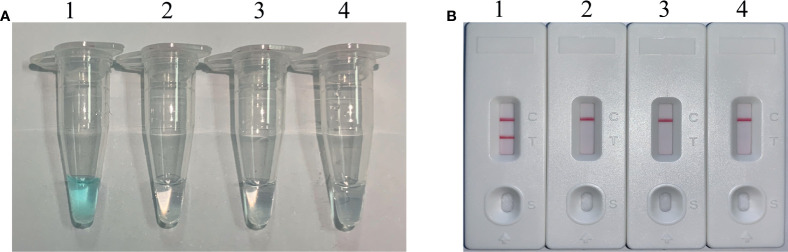
Identification and confirmation of *H*. *influenzae*-MCDA products. **(A)** The visible color changes of amplification products of *H*. *influenzae*-MCDA assay were analyzed by VDR. **(B)** The products of *H*. *influenzae*-MCDA were visually detected with Lateral flow biosensor. Tube 1/biosensor 1, positive amplification of *H*. *influenzae* strain; tube 2/biosensor 2, negative control of *Staphylococcus aureus* strain; tube 3/biosensor 3, negative control of *Escherichia coli* strain; tube 4/biosensor 4, blank control (double-distilled water, DW).

To verify the specificity and feasibility of *H. influenzae* MCDA primers, *H. influenzae* MCDA assays were performed at 63°C for 1 h, with the genomic DNA templates from pure cultures. By adding the Visual Detection Reagent (VDR) into the amplification mixtures, the MCDA reaction result could be directly detected with the naked eyes, and the positive result was presented from colorless to blue (**Figure 2A**). With LFB, the two red bands appeared on the biosensors, involving test line (TL) and control line (CL), which were on behalf of the positive results. While, the single red band presented on the biosensors on behalf of negative and blank controls (**Figure 2B**). As shown in **Figure 2**, the positive results were generated with templates from *H. influenzae* strains, but not with templates from non-*H. influenzae* strains as well as the blank control. Therefore, these results showed that the primer set was suitable for the establishment of the MCDA-LFB method for *H. influenzae* detection.

### The Optimal Amplification Temperature for *H. influenzae-*MCDA Assay 

**Figure 3:**
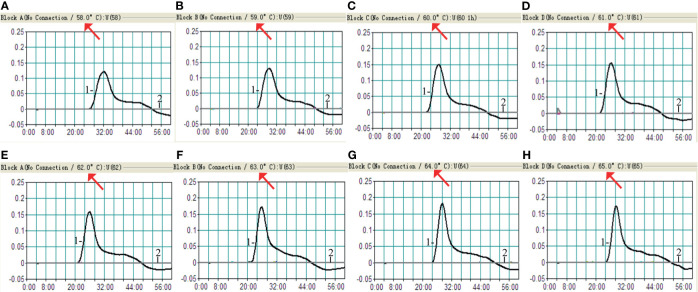
Amplification temperature optimization for *H. influenzae*-MCDA assay. The MCDA reaction for detection of *H. influenzae* was monitored by real-time turbidimeter, and the corresponding curves of concentrations of DNA were showed in the pictures. The threshold value was 0.1, and the turbidity of > 0.1 was considered positive. Eight kinetic graphs **(A–H)** were acquired at a series of temperatures from 5 to 65°C (with 1°C intervals) with DNA templates of *H. influenzae* at the level of 1 pg per reaction. Signal 1, positive amplification of *H. influenzae* strain; signal 2, negative control of *Staphylococcus aureus* strain.

The optimum temperature for the *H. influenzae-*MCDA-LFB assay, during the amplification process, was detected with 1 pg *H. influenzae* genomic DNA in per reaction, and at temperatures of 58–65°C for 1 h by observing turbidity continuously. The amplification of OMP *P6* was detected at all tested temperatures, but the threshold value of absorbance (0.1) was reached most quickly at 61°C (**Figure 3**). Moreover, after incubation of 1 h, there was no non-specific amplification in the negative control. Therefore, the 61°C served as the optimal temperature for the subsequent MCDA-LFB tests conducted in this study.

### Sensitivity of *H. influenzae*-MCDA-LFB Assay

**Figure 4:**
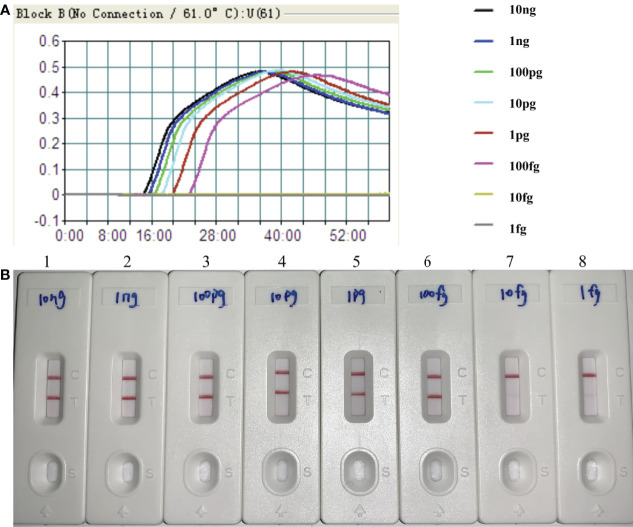
Sensitivity of *H*. *influenzae-*MCDA-LFB assay with serially diluted genomic DNA templates. Two measurement assays, namely, real-time turbidity **(A)** and later flow biosensor **(B)**, were applied for detecting MCDA products. The serial dilutions of target DNA templates (10 ng, 1 ng, 100 pg, 10 pg, 1 pg, 100 fg, 10 fg, and 1 fg) were used for sensitivity analysis. Turbidity signals **(A)** / Biosensors **(B)** 1–8, respectively, represent the DNA levels of 10 ng, 1 ng, 100 pg, 10 pg, 1 pg, 100 fg, 10 fg, and 1 fg per reaction. The DNA levels of 10 ng, 1 ng, 100 pg, 10 pg, 1 pg, 100 fg per reaction generated the positive reactions.

By the MCDA-LFB method, the sensitivity of *H. influenzae*-MCDA-LFB assay was tested with serial dilutions of the extracted DNA of *H. influenzae.* The final concentrations of DNA templates were 10 ng, 1 ng, 100 pg, 10 pg, 1 pg, 100 fg, 10 fg, and 1 fg in per reaction mixture. The experimental LoD of *H. influenzae-*MCDA-LFB method was as little as 100 fg in pure culture in **Figure 4**.

### Specificity of *H. influenzae*-MCDA-LFB Assay

**Figure 5:**
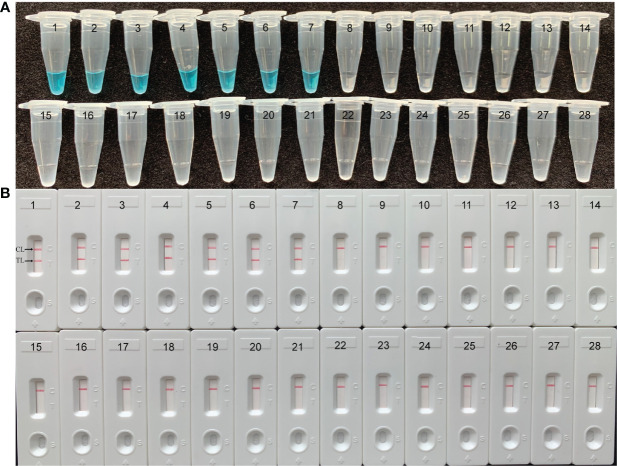
Specificity of *H*. *influenzae*-MCDA-LFB assay with DNA templates from distinct strains. The MCDA reactions were conducted with different genomic DNA templates and were analyzed by means of visual mode including VDR **(A)** and later flow biosensor **(B)**. 1–7, *H*. *influenzae* strains; 8–9, *H*. *parainfluenzae*; 10, *H*. *haemolyticus*; 11, *H. parahaemolyticus*; 12, Enteroadhesive *E*. *coli*;13, Enterohemorrhagic *E*. *coli*; 14, Enteropathogenic *E*. *coli*; 15, Enteroinvasive *E*. *coli*; 16, Enterotoxigenic *E*. *coli*; 17–18, *Listeria monocytogenes*; 19–20, *Pseudomonas aeruginosa*; 21, *Streptococcus suis*; 22–23, *Klebsiella pneumoniae*; 24, *Staphylococcus aureus*; 25, *Staphylococcus epidermidis*; 26, *Staphylococcus haemolyticus*; 27, *Acinetobacter baumannii*; 28, *Enterococcus faecalis*.

The genomic DNA templates of each pathogen, roughly 10 ng, extracted from 7 *H. influenzae* strains, 17 non-*H. influenzae* strains, 2 *H. parainfluenzae* strains, 1 *H. haemolyticus* strain, and 1 *H. parahaemolyticus* strain were applied to verify the specificity of *H. influenzae*-MCDA-LFB assay. The positive amplification was acquired from all the *H. influenzae* isolates, along with two red lines (TL and CL) presented on the biosensors (**Figure 5**). In contrast, only a single red line (CL) presented on the biosensor, indicating the negative results for non-*H. influenzae* isolates and other *Haemophilus* species (**Figure 5**).

### Application of MCDA-LFB to *H. influenzae* Clinical Specimens

**Figure 6:**
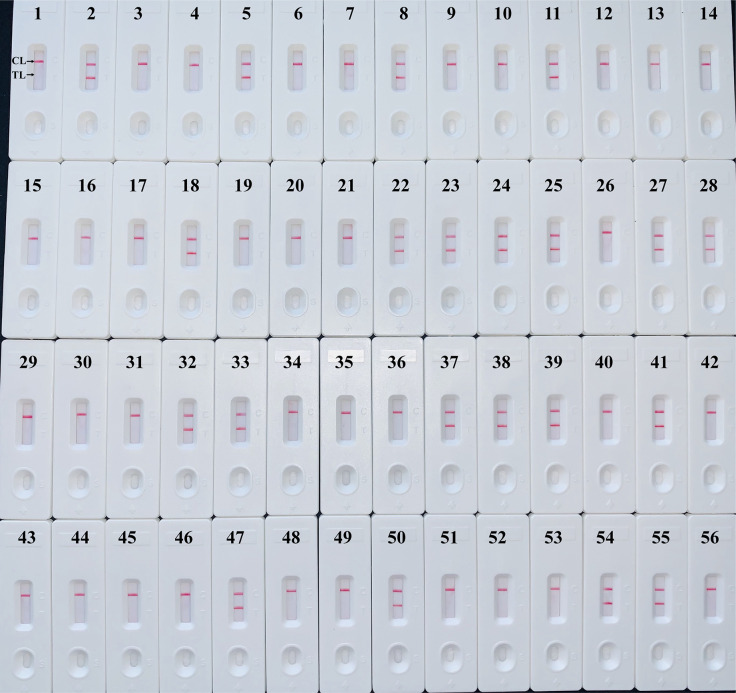
Sensitivity of MCDA-LFB assay for detecting *H. influenzae* in clinical samples. Later flow biosensor was applied for detecting MCDA amplicons. The numbers 2, 5, 8, 11, 18, 22–25, 27, 28, 32, 33, 37–39, 41, 47, 50, 54, 55 represented the positive results. Other numbers represented the negative results.

**Figure 7:**
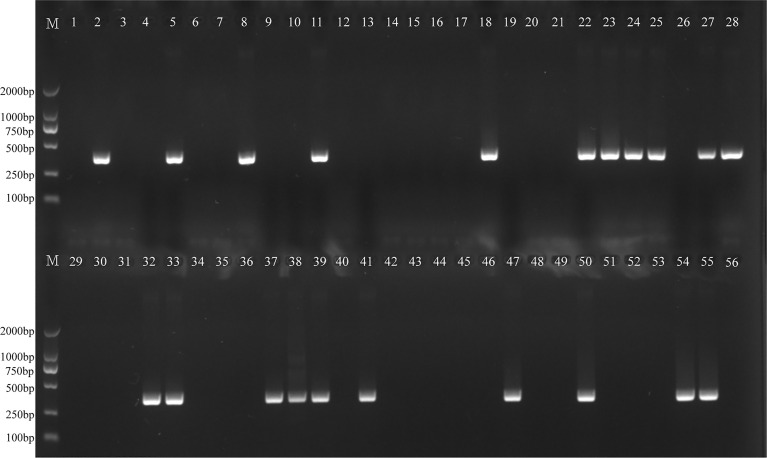
PCR assay for detecting *H. influenzae* in clinical samples. The numbers 2, 5, 8, 11, 18, 22–25, 27, 28, 32, 33, 37–39, 41, 47, 50, 54, 55 represented the positive results. Other numbers represented the negative results.

The *H. influenzae-*MCDA-LFB assay and PCR detection were used to detect *H. influenzae* from 56 randomly mixed DNA templates as previously introduced extracted from sputum samples. One μl DNA template of each sample was amplified in PCR detection and *H. influenzae*-MCDA test, respectively. Each test was repeated at least twice, and then 0.5 μl amplification products of *H. influenzae*-MCDA test were detected by LFB. Both *H. influenzae-*MCDA-LFB (**Figure 6**) and PCR (**Figure 7**) results showed that 21 of 56 sputum samples were *H. influenzae* positive, completely in accordance with traditional cultivation results.

## Discussion

*H. influenzae* is an important human pathogen that is relative to meningitis, bacteremia, and otitis media (Torigoe et al., 2007). Herein, an MCDA-LFB method according to the OMP *P6* gene was successfully developed in order to identify all *H. influenzae* strains. By means of MCDA-LFB method, a total of 10 specific primers were designed to recognize 10 regions of the target sequence (**Figure 1**). With the genomic DNA templates extracted from 7 *H. influenzae* strains, 17 non-*H. influenzae* strains, 2 *H. parainfluenzae* strains, 1 *H. haemolyticus* strain, and 1 *H. parahaemolyticus* strain, the specificity of MCDA-LFB was successfully assessed. Obviously, the positive results were acquired from the *H. influenzae* strains but not from non-*H. influenzae* strains and other *Haemophilus* species (**Figure 5**). In our study, the MCDA-LFB method was highly specific to *H. influenzae* rather than non-*H. influenzae* strains and other *Haemophilus* species. Thus, this method could be a worthy tool for identifying target pathogen. 

It could not be ignored that the necessary sample collection and DNA separation steps that took place upstream of MCDA-LFB might took some time. However, the extraction of DNA could be quickly finished with kits. In MCDA-LFB assay, the advantages were in the downstream steps, and the difference from the traditional PCR method was that the thermal denature and the changes were not required. The requirement of complicated equipment was also reduced. Besides, the incubation temperature of MCDA-LFB method was only at 61°C, which simplified the reaction process (Wang et al., 2015). After the MCDA amplification, the white precipitate would be produced by magnesium pyrophoshate, which could be detected by the naked eye after adding colorimetric indicator (HNB or FD) in natural light (Zhang et al., 2014). Another way, by adding dyes such as SYBR Green I or calcein dyes, and with UV irradiation, the amplification products could be detected by agarose gel electrophoresis or measured turbidity with spectrophotometric instrument (Zhang et al., 2014). In our study, the MCDA products could be detected in an objective and visual way within 2 min, with the simple biosensor (LFB) loaded in a plastic device. So, the *H. influenzae*-MCDA-LFB method could be used as a rapid, simple, and nearly instrument-free technology platform for detecting target bacterium. However, the limitations of MCDA assay were as follows: First of all, due to a set of 10 primers, the MCDA nucleic acid products were a complex mixture; therefore, these product analysis techniques cannot distinguish between specific and non-specific amplifications (Zhang et al., 2014). Secondly, with the naked eye, it is difficult to avoid assessing the color or turbidity subjectively, and it is also possible that if the concentration of target templates in the reaction is very low, the sample may be a little ambiguous to the unaided eyes. Thirdly, additional procedures, such as gel electrophoresis and turbidity detection, are required to realize surveillance technology, which may limit the application of MCDA assay.

In this study, the LoD of *H. influenzae*-MCDA-LFB assay was as few as 100 fg per reaction in pure culture, which is more sensitive than that of traditional *H. influenzae*-PCR method. In addition, the detection result of clinical sputum samples also showed the better sensitivity and less time-consuming of MCDA-LFB (**Figure 6**), which was consistent with the traditional culture method and PCR detection.

In conclusion, we successfully established the available MCDA-LFB method for detection of *H. influenzae* targeting OMP *P6* gene in pure cultures and clinical sputum samples. High selectivity for detection of target pathogen, high sensitivity of 100 fg per reaction in pure culture, and higher sensitivity and less time-spending in the detection of clinical samples were revealed with this method. The procedure of MCDA-LFB was simple, rapid, and sensitive, which was with no need for specialized equipment. Consequently, the MCDA-LFB assay will offer a feasible strategy for rapid detection of the pathogenic bacterium and is suitable for application in resource-limited situations.

## Data Availability Statement

The raw data supporting the conclusions of this article will be made available by the authors, without undue reservation.

## Ethics Statement

Our study was approved by the Ethical Committee of Dingzhou People’s hospital, and guarduans of the enrolled patients signed informed consent documents.

## Author Contributions

QLC, SSL, XLC, and YG conceived and designed the experiments. QC, SSL, and LW performed the experiments. SSL, LW, JC, MYL, and SPL contributed the reagents and materials. QC and SSL analyzed the data and wrote the paper. All authors contributed to the article and approved the submitted version.

## Conflict of Interest

Authors QLC, SSL and LW were employed by company Qingdao Haier Biotech Co. Ltd.

The remaining authors declare that the research was conducted in the absence of any commercial or financial relationships that could be construed as a potential conflict of interest.

## Publisher’s Note

All claims expressed in this article are solely those of the authors and do not necessarily represent those of their affiliated organizations, or those of the publisher, the editors and the reviewers. Any product that may be evaluated in this article, or claim that may be made by its manufacturer, is not guaranteed or endorsed by the publisher.

## References

[B1] Barbour M. L. (1996). Conjugate Vaccines and the Carriage of *Haemophilus Influenzae* Type B. Emerg. Infect. Dis. 2 (3), 176–182. doi: 10.3201/eid0203.960303 8903227 PMC2626802

[B2] Brown P. D. Lerner S. A. (1998). Community-Acquired Pneumonia. Lancet 352 (9136), 1295–1302. doi: 10.1136/adc.85.6.445 9788476

[B3] Corless C. E. Guiver M. Borrow R. Edwards-Jones V. Fox A. J. Kaczmarski E. B. (2001). Simultaneous Detection of *Neisseria Meningitidis*, *Haemophilus Influenzae*, and *Streptococcus Pneumoniae* in Suspected Cases of Meningitis and Septicemia Using Real-Time PCR. J. Clin. Microbiol. 39 (4), 1553–1558. doi: 10.1128/JCM.39.4.1553-1558.2001 11283086 PMC87969

[B4] Deich R. A. Metcalf B. J. Finn C. W. Farley J. E. Green B. A. (1988). Cloning of Genes Encoding a 15,000-Dalton Peptidoglycan-Associated Outer Membrane Lipoprotein and an Antigenically Related 15,000-Dalton Protein From *Haemophilus Influenzae* . J. Bacteriol. 170 (2), 489–498. doi: 10.1128/jb.170.2.489-498.1988 2828309 PMC210680

[B5] Falla T. J. Crook D. W. Brophy L. N. Maskell D. Kroll J. S. Moxon E. R. (1994). PCR for Capsular Typing of *Haemophilus Influenzae* . J. Clin. Microbiol. 32 (10), 2382. doi: 10.1002/jctb.280610214 7814470 PMC264070

[B6] Hasegawa K. Chiba N. Kobayashi R. Murayama S. Y. Ubukata K. (2004). Rapidly Increasing Prevalence of β-Lactamase-Nonproducing, Ampicillin-Resistant Haemophilus Influenzae Type B in Patients With Meningitis. Antimicrob. Agents Ch. 48 (5), 1509–1514. doi: 10.1128/AAC.48.5.1509-1514.2004 PMC400528 15105098

[B7] Hendolin P. H. Markkanen A. Ylikoski J. Wahlfors J. J. (1997). Use of Multiplex PCR for Simultaneous Detection of Four Bacterial Species in Middle Ear Effusions. J. Clin. Microbiol. 35 (11), 2854–2858. doi: 10.1128/JCM.35.11.2854-2858.1997 9350746 PMC230074

[B8] Hotomi M. Tabata T. Kakiuchi H. Kunimoto M. (1993). Detection of Haemophilus Influenzae in Middle Ear of Otitis Media With Effusion by Polymerase Chain Reaction. Int. J. Pediatr. Otorhi. 27 (2), 119–126. doi: 10.1016/0165-5876(93)90127-O 8258479

[B9] Howie V. M. Ploussard J. H. Lester R. L. (1970). Otitis Media: A Clinical and Bacteriologic Correlation. Pediatrics 45 (1), 29–35. doi: 10.1203/00006450-197011000-00015 4903112

[B10] Karalus R. J. Murphy T. F. (1999). Purification and Characterization of Outer Membrane Protein P6, A Vaccine Antigen of Non-Typeable Haemophilus Influenzae. FEMS Immunol. Med. Mic. 26 (2), 159–166. doi: 10.1111/j.1574-695X.1999.tb01384.x 10536303

[B11] Kim D. W. Kilgore P. E. Kim E. J. Kim S. A. Anh D. D. Seki M. (2011). Loop-Mediated Isothermal Amplification Assay for Detection of *Haemophilus Influenzae* Type B in Cerebrospinal Fluid. J. Clin. Microbiol. 49 (10), 3621–3626. doi: 10.1128/JCM.00515-11 21832019 PMC3187296

[B12] Li S. Jiang W. Huang J. Liu Y. Ren L. Zhuang L.. (2020). Highly Sensitive and Specific Diagnosis of Coronavirus Disease 19 (COVID-19) by Reverse Transcription Multiple Cross Displacement Amplification-Labelled Nanoparticles Biosensor. Eur. Respir. J. 56, 2002060. doi: 10.1183/13993003.02060-2020 32859676 PMC7453731

[B13] Lu J. J. Perng C. L. Lee S. Y. Wan C. C. (2000). Use of PCR With Universal Primers and Restriction Endonuclease Digestions for Detection and Identification of Common Bacterial Pathogens in Cerebrospinal Fluid. J. Clin. Microbiol. 38 (6), 2076–2080. doi: 10.1128/JCM.38.6.2076-2080.2000 10834956 PMC86732

[B14] Murphy T. F. Bartos L. C. Campagnari A. A. Nelson M. B. Apicella M. A. (1986). And Antigenic Characterization of the P6 Protein of Nontypable *Haemophilus Influenzae* . Infect. Immun. 54 (3), 774–779. doi: 10.1128/IAI.54.3.774-779.1986 PMC260236 3491049

[B15] Murphy T. F. Nelson M. B. Dudas K. C. Mylotte J. M. Apicella M. A. (1985). Identification of a Specific Epitope of Haemophilus Influenzae on a 16,600-Dalton Outer Membrane Protein. J. Infect. Dis. 152 (6), 1300–1307. doi: 10.1093/infdis/152.6.1300 2415644

[B16] Nelson M. B. Apicella M. A. Murphy T. F. Vankeulen H. Spotila L. D. Rekosh D. (1988). Cloning and Sequencing of Haemophilus Influenzae Outer Membrane Protein P6. Infect. Immun. 56 (1), 128–134. doi: 10.1128/IAI.56.1.128-134.1988 3257200 PMC259246

[B17] Nelson M. B. Munson R. S. Apicella M. A. Sikkema D. J. Molleston J. P. Murphy T. F. (1991). Molecular Conservation of the P6 Outer Membrane Protein Among Strains of *Haemophilus Influenzae*: Analysis of Antigenic Determinants, Gene Sequences, and Restriction Fragment Length Polymorphisms. Infect. Immun. 59 (8), 2658–2663. doi: 10.1128/IAI.59.8.2658-2663.1991 1713197 PMC258070

[B18] Peltola H. (2000). Worldwide Haemophilus Influenzae Type B Disease at the Beginning of the 21st Century: Global Analysis of the Disease Burden 25 Years After the Use of the Polysaccharide Vaccine and a Decade After the Advent of Conjugates. Clin. Microbiol. Rev. 13 (2), 302–317. doi: 10.1128/cmr.13.2.302-317.2000 10756001 PMC100154

[B19] Torigoe H. Seki M. Yamashita Y. Sugaya A. Maeno M. (2007). Detection of Haemophilus Influenzae by Loop-Mediated Isothermal Amplification (LAMP) of the Outer Membrane Protein P6 Gene. Jpn. J. Infect. Dis. 60 (1), 55–58. 17314429

[B20] Ueyama T. Kurono Y. Shirabe K. Takeshita M. Mogi G. (1995). High Incidence of Haemophilus Influenzae in Nasopharyngeal Secretions and Middle Ear Effusions as Detected by PCR. J. Clin. Microbiol. 33 (7), 1835–1838. doi: 10.1128/JCM.33.7.1835-1838.1995 7665655 PMC228280

[B21] van Ketel R. J. Wever B. D. Alphen L. V. (1990). Detection of *Haemophilus Influenzae* in Cerebrospinal Fluids by Polymerase Chain Reaction DNA Amplification. J. Med. Microbiol. 33 (4), 271–276. doi: 10.1099/00222615-33-4-271 2258914

[B22] Wang Y. Li H. Li D. Li K. Wang Y. Xu J.. (2016). Multiple Cross Displacement Amplification Combined With Gold Nanoparticle-Based Lateral Flow Biosensor for Detection of *Vibrio Parahaemolyticus* . Front. Microbiol. 7, 2047. doi: 10.3389/fmicb.2016.02047 28066368 PMC5177632

[B23] Wang Y. Li H. Wang Y. Xu H. Xu J. Ye C. (2018). Antarctic Thermolabile Uracil-DNA-Glycosylase-Supplemented Multiple Cross Displacement Amplification Using a Label-Based Nanoparticle Lateral Flow Biosensor for the Simultaneous Detection of Nucleic Acid Sequences and Elimination of Carryover Contamination. Nano Res. 11 (5), 2632–2647. doi: 10.1007/s12274-017-1893-z

[B24] Wang Y. Wang Y. Luo L. Liu D. Luo X. Xu Y.. (2015). Rapid and Sensitive Detection of Shigella Spp. And *Salmonella* Spp. By Multiple Endonuclease Restriction Real-Time Loop-Mediated Isothermal Amplification Technique. Front. Microbiol. 6, 1400. doi: 10.3389/fmicb.2015.01400 26697000 PMC4677097

[B25] Zhang X. Lowe S. B. Gooding J. J. (2014). Brief Review of Monitoring Methods for Loop-Mediated Isothermal Amplification (LAMP). Biosens. Bioelectron. 61, 491–499. doi: 10.1016/j.bios.2014.05.039 24949822

